# The Simultaneous Determination of Six Flame Retardants in Water Samples Using SPE Pre-concentration and UHPLC-UV Method

**DOI:** 10.1007/s11270-014-1866-4

**Published:** 2014-02-12

**Authors:** Bartosz Kowalski, Maciej Mazur

**Affiliations:** Department of Inorganic, Analytical and Electrochemistry, Chemical Faculty, The Silesian University of Technology, 7 Marcina Strzody Str, 44-100 Gliwice, Poland

**Keywords:** Flame retardants, Water samples, Ultra HPLC, SPE columns

## Abstract

Analytical method for the determination of six flame retardants (FRs) from two groups was proposed. These groups included the brominated flame retardants (BFRs) 3,3′,5,5′-tetrabromobisphenol A (TBBPA), 1,2,5,6,9,10-hexabromocyclododecane (HBCD) and tetrabromophthalic anhydride (TBPA) and triester organophosphate flame retardants (OPFRs) tris(2,3-dibromopropyl) phosphate (TBPP), ethylhexyl diphenyl phosphate (EHDP) and triphenyl phosphate (TPhP). Reversed phase ultrahigh-performance liquid chromatography (UHPLC) with a UV detector, different chromatographic columns, different mobile phases and gradient elution programmes were used to obtain the best separations within the shortest possible time. Solid-phase extraction (SPE) was examined as a pre-concentration step from distilled water. The column with the highest recoveries (the Bond Elut ENV column gave recoveries over 70 % for all compounds) was then tested on 1-L blank surface water samples. The proposed analytical procedure was applied for the determination of FRs in surface water samples. The concentrations of FRs found in water samples ranged from 0.03 (TPhP) to 3.10 μg L^−1^ (HBCD). Method detection limits (MDLs) ranged from 0.008 to 0.518 μg L^−1^, and method quantification limits (MQLs) ranged from 0.023 to 1.555 μg L^−1^ for all compounds.

## Introduction

Flame retardants (FRs) are synthetic additives that are widely used in a variety of commercial products such as electrical and electronic equipment, plastics, textiles, furniture and many others (Guerra et al. 2011). Organic FRs can be divided into two groups: halogenated flame retardants (mostly brominated FRs (BFRs)) and organophosphate flame retardants (OPFRs). The most frequently used BFRs are polybrominated diphenylethers (PBDEs), tetrabromobisphenol A (TBBPA) and hexabromocyclododecane (HBCD) (Lopez et al. 2011). The usage of FRs has been growing rapidly in recent years. BFRs are the most often used FRs, and their market is still growing. However, the estimated annual usage of OPFRs was almost twice as that of all BFRs combined in Western Europe (Reemtsma et al. 2008). Many FRs have been banned for use due to their potential toxicity, environmental occurrence and accumulation in human tissues (Lopez et al. 2011). FRs taken off the market are likely to be replaced by others. Though, in recent years, the REACH regulatory has been introduced in Europe to improve the protection of human health and environment, it is still necessary to monitor the FRs in environmental samples.

The majority of available FR studies used either high-performance liquid chromatography (HPLC) or gas chromatography (GC) as the instrumental analysis tool for the determination of FRs in water samples. HPLC technique was used mostly with MS or tandem MS/MS detector (Mascolo et al. 2010; Rodil et al. 2009; Wang et al. 2011; Wu et al. 2009; Quintana and Reemtsma 2006; Bacaloni et al. 2009). Only one publication found described the determination of FRs (only those included in this study) in water samples using HPLC and UV detector (Yu and Hu 2007). In addition, GC technique was used with MS or MS/MS detectors (Trenholm et al. 2008; Yan et al. 2012; Sanchez-Brunete et al. 2009; Cristale et al. 2012; Chung and Ding 2009; Garcia-Lopez et al. 2009), and only few studies used NPD (Garcia-Lopez et al. 2007; Rodriguez et al. 2006) and FPD (Gao et al. 2013) detectors. As a pre-concentration step, most of the authors used solid-phase extraction (SPE) with different sorbents. However, some other extraction techniques as PLE (Mascolo et al. 2010), MASE (Quintana and Reemtsma 2006), DLLME (Garcia-Lopez et al. 2007) and SPME (Gao et al. 2013) were also applied.

The aim of this work was the development of an analytical procedure for the extraction and simultaneous determination of selected FRs. For the extraction, the SPE method with different sorbents was evaluated, and for the determination, the ultrahigh-performance liquid chromatography (UHPLC)-UV method was developed. In this paper, we focused on three BFRs that are widely used and produced in high amounts, namely, TBBPA, HBCD and tetrabromophthalic anhydride (TBPA), and three OPFRs, namely, tris(2,3-dibromopropyl) phosphate (TBPP), ethylhexyl diphenyl phosphate (EHDP) and triphenyl phosphate (TPhP). The selection of OPFRs for this study was justified by the fact that there are few data available on environmental contamination with these compounds (Chen et al. 2012).

## Materials and Methods

### Chemical and Reagents

All flame retardants were purchased from Sigma-Aldrich (Poznan, Polska); HPLC grade water, methanol and acetonitrile were acquired from Merck (Darmstadt, Germany), and analytical grade methanol (MeOH), acetonitrile (ACN) and ethyl acetate (EtAc) were bought from POCH (Gliwice, Poland).

Stock solutions (1 mg mL^−1^) of HBCD, TBBPA, TBPA and TPhP were prepared by dissolving 10 mg of the appropriate standard in 10 mL of acetonitrile (HPLC grade). The stock solutions (1 mg mL^−1^) of EHDP and TBPP were prepared by dilution of the appropriate volume of standard (10 mg mL^−1^) in acetonitrile (HPLC grade) to the final concentration. Stock solutions of all FRs were stable for over 3 months at 4 °C. Working solutions were prepared daily by mixing the appropriate volume of each stock solution with acetonitrile (analytical grade).

### Instrumentation

The ultra-HPLC system included two L-2160U pumps (LaChrom Elite, Merck Hitachi), a L-2350 column oven, a L-2200U autosampler and a L-2400U UV detector (LaChrom Ultra, Merck Hitachi). The data were collected with EZChrom Elite software. Analytical, reversed phase columns Chromolith® Fast Gradient monolithic C_18_e column (50 mm × 2 mm) from Merck, Hypersil GOLD™ (50 mm × 2.1 mm, 1.9 μm) from Thermo Fisher Scientific Inc. and Hibar® HR Purospher® STAR RP-18e (50 mm × 2.1 mm, 2 μm) from Merck were tested. The SPE was performed using J.T. Baker spe-12G (Deventer, Netherlands).

### The SPE Procedures

Several SPE columns were tested using the same sample extraction procedure for the water samples. These columns included Bond Elut columns (Varian): an ENV column (6 mL, 500 mg), a NEXUS column (6 mL, 200 mg, Varian), a PPL column (6 mL, 500 mg) and a C18LO column (6 mL, 1 g); an Oasis HLB column (6 mL, 500 mg, Waters); and an extraction disc BAKERBOND® Speedisk® C18 (50 mm). Each column (with the exception of Speedisk®) was conditioned with 6 mL of ethyl acetate and 6 mL of distilled water at a flow rate of 1 mL min^−1^. Subsequently, 1 L of distilled water spiked with all tested compounds was passed through at a flow rate of 10 mL min^−1^. The column was dried for 5 min, and then the analytes were eluted with 2 × 5 mL of ethyl acetate, evaporated to dryness under a gentle nitrogen stream and reconstituted in 1 mL acetonitrile (analytical grade). Then, 2 μL of the obtained extracts was injected through the autosampler into the ultra-HPLC system. The columns with the highest obtained recoveries were then tested on 1 L surface water.

### Chromatographic Separations

Different mobile phases were tested for the determination of flame retardants on three analytical columns at a temperature of 25 ºC using ultra-HPLC equipment and the UV detector. A gradient comprised of two solvents was tested each time, where solvent A was acetonitrile or methanol and B was pure water. The solvent gradient was optimised to obtain the best separations in the shortest possible time on each column. The column eluent was detected and quantitated at the characteristic detection wavelength for each flame retardant using the UV detector.

## Results and Discussion

### Development of Chromatographic System

A two-solvent gradient elution programme was used to obtain the best separations for all tested flame retardants. Several systems were examined using different analytical columns and different eluents. The best separations on each analytical column were obtained with the mobile phase comprised of acetonitrile and water. When using methanol and water as a mobile phase, higher retention times were achieved for each flame retardant.

The best gradient elution programme for the Chromolith® Fast Gradient monolithic C_18_e column (50 mm × 2 mm) did not gave satisfactory separations; however, the short analysis time (2 min) was achieved.

**Fig. 1:**
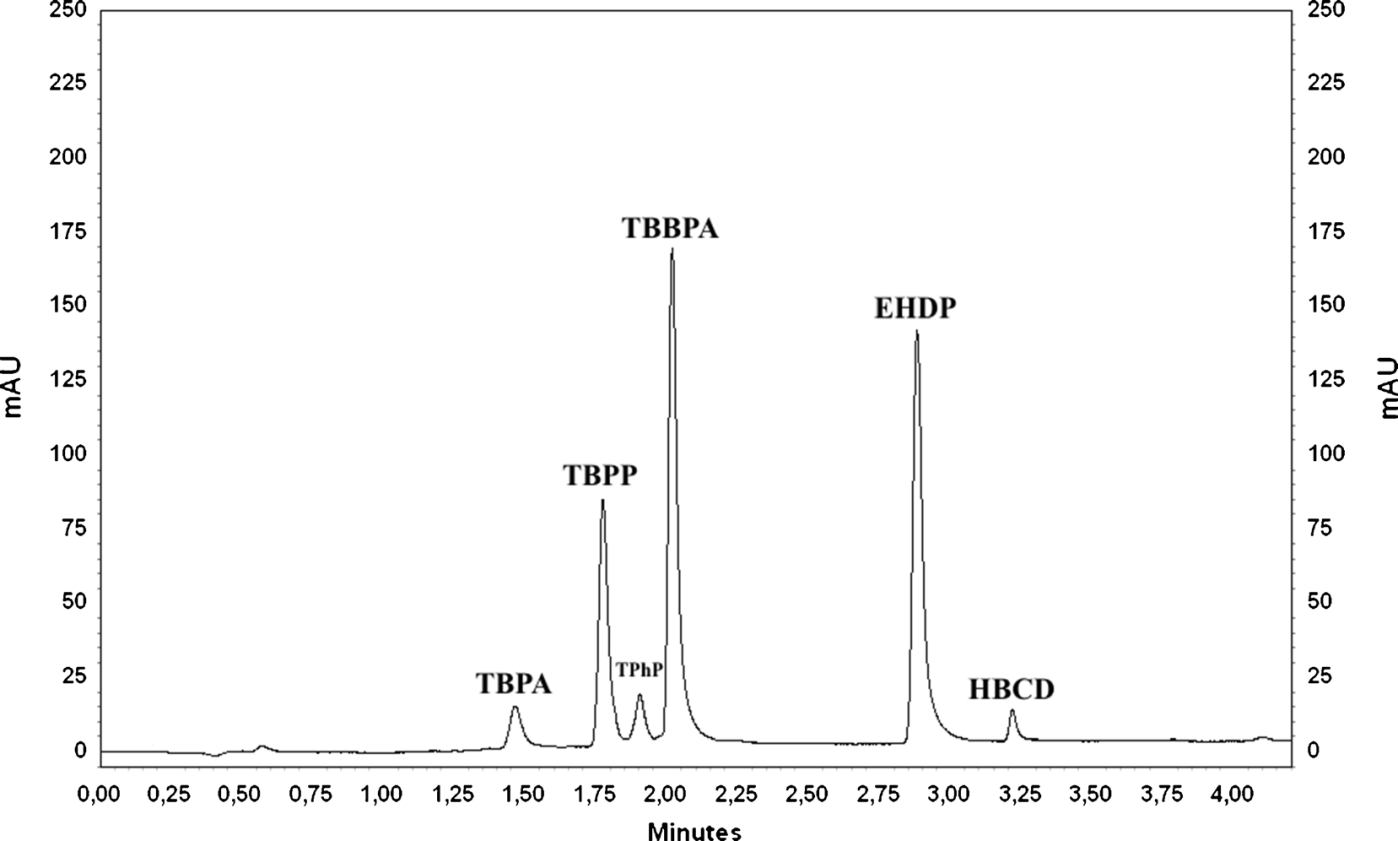
The chromatogram of standard mixture containing 2 μg mL^−1^ for all FRs performed on the UV detector

The next tested analytical column was Hypersil GOLD™ (50 mm × 2.1 mm, 1.9 μm). The gradient elution programme was modified and set for the determination of flame retardants (Table 1). The separations of all compounds were satisfactory, and the gradient elution programme allows the use of a short analysis time (less than 3.5 min) (Fig. 1).

**Table 1:** The best gradient elution programme for Hypersil GOLD

Time [min]	Solvent		Flow rate [mL min^−1^]
	A^a^ [%]	B^b^ [%]	
0.0	50	50	0.5
1.0	70	30	0.5
2.0	90	10	0.5
3.0	100	0	0.5
4.0	100	0	0.5

^a^Acetonitrile

^b^Water

**Fig. 2:**
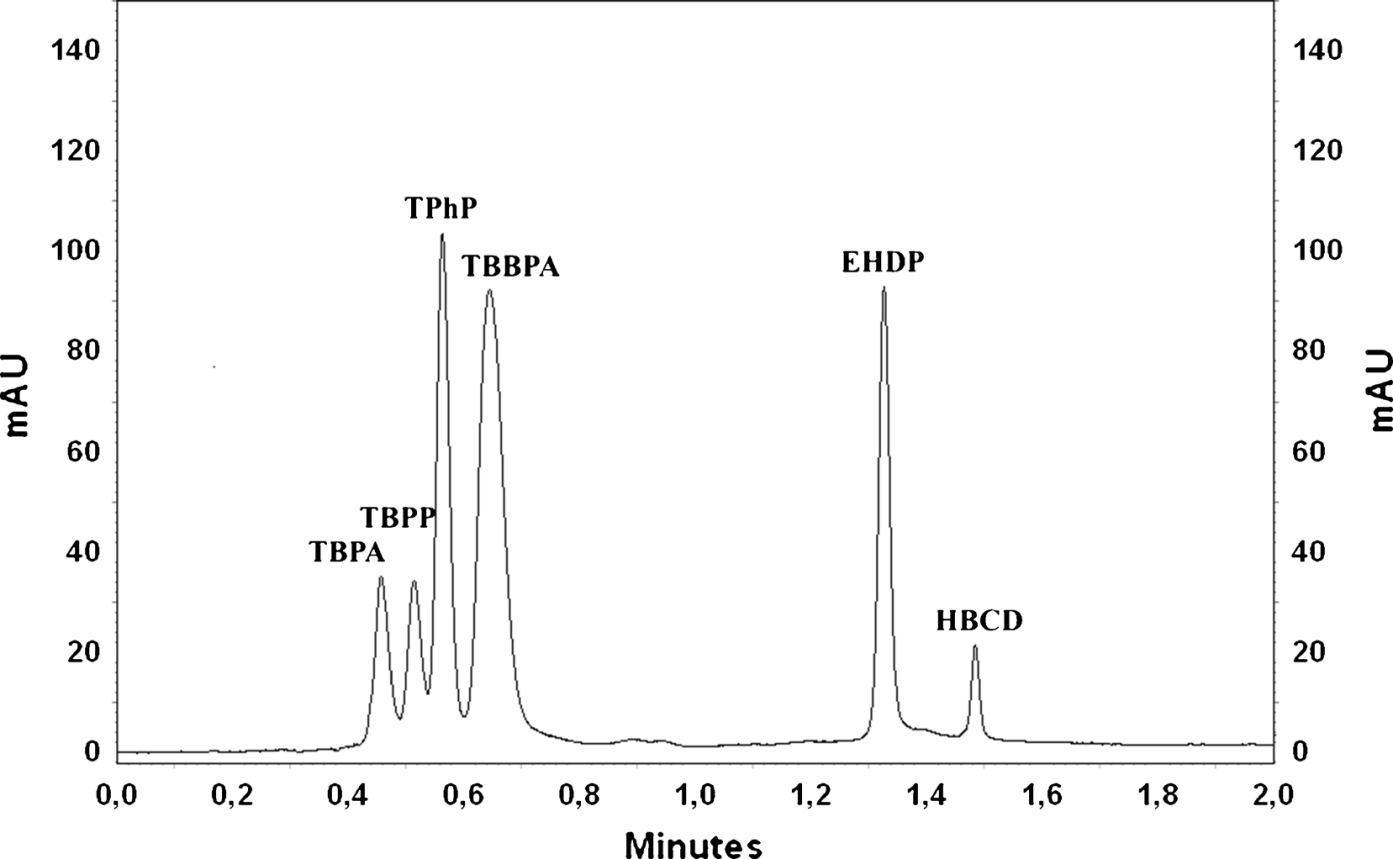
The chromatogram of standard mixture containing 10 μg mL^−1^ for all FRs performed on the UV detector

The same mobile phase was used for the last tested analytical column Hibar® HR Purospher® STAR RP-18e (50 mm × 2.1 mm, 2 μm). Several modifications, such as a slightly increased flow rate, were introduced to achieve good separations within shorter analysis time (Table 2). All flame retardants were separated satisfactorily over the course of a 2-min analysis (Fig. 2).

**Table 2:** The best gradient elution programme for Hibar®

Time [min]	Solvent		Flow rate [mL min^−1^]
	A^a^ [%]	B^b^ [%]	
0.0	70	30	0.7
1.0	100	0	0.7
2.0	100	0	0.7

^a^Acetonitrile

^b^Water

The following gradient was chosen for the determination of FR in water samples and to obtain recovery levels on each tested SPE column. Analytical wavelengths, retention times and coefficients of variation (CV) are shown in Table 3. The short analysis time allowed examination of a great number of samples in a single day, which can save expensive HPLC grade chemicals and reduce labour costs.

**Table 3:** Wavelengths, retention times and coefficient of variation (*n* = 6)

Drug	Wavelength [nm]	Retention time [min]	Coefficient of variation [%]
TBPA	253	0.455	0.36
TBPP	205	0.518	0.80
TPhP	205	0.567	0.98
TBBPA	205	0.665	0.92
EHDP	205	1.324	0.62
HBCD	205	1.496	0.54

### Recovery

Different SPE columns were tested using distilled water spiked with 2 μg of TPhP, TBBPA and EHDP and 5 μg of TBPA, TBPP and HBCD. First samples (100 mL) were passed through each extraction column. Three eluents were used (methanol, acetonitrile and ethyl acetate) to examine the recoveries for all flame retardants. The recoveries obtained using the extraction procedure (2.3) are presented in Table 4.

**Table 4:** Recoveries for FRs using three eluents (*n* = 3)

Extraction column	Eluent	Recoveries [%] (% RSD)					
		TBPA	TBPP	TPhP	TBBPA	EHDP	HBCD
Oasis HLB	MeOH	–	80 (6.0)	39 (8.9)	<10	16 (6.0)	<10
	EtAc	–	82 (6.0)	62 (4.5)	18 (8.6)	21 (3.5)	32 (10)
	ACN	–	78 (4.2)	38 (7.1)	<10	15 (6.3)	17 (7.5)
Bond Elut NEXUS	MeOH	–	47 (7.6)	58 (6.3)	30 (7.9)	17 (10)	<10
	EtAc	–	52 (8.3)	76 (9.6)	32 (11)	22 (11)	25 (7.5)
	ACN	–	49 (7.1)	68 (9.4)	18 (8.1)	20 (7.9)	22 (6.1)
Bond Elut ENV	MeOH	–	79 (7.6)	11 (2.6)	27 (11)	<10	<10
	EtAc	–	91 (9.4)	89 (5.7)	69 (4.0)	84 (8.4)	77 (10)
	ACN	–	69 (5.1)	79 (3.8)	35 (4.6)	24 (7.8)	25 (7.6)
Bond Elut C18LO	MeOH	–	83 (9.6)	90 (5.6)	34 (10)	55 (5.6)	46 (7.8)
	EtAc	–	95 (6.9)	92 (5.7)	56 (8.5)	75 (8.4)	75 (5.7)
	ACN	–	93 (6.8)	89 (4.1)	11 (7.2)	49 (5.8)	48 (10)
Bond Elut PPL	MeOH	–	97 (7.4)	27 (10)	33 (9.2)	13 (11)	<10
	EtAc	–	99 (10)	85 (5.5)	79 (6.2)	92 (3.2)	68 (8.1)
	ACN	–	90 (6.4)	83 (7.0)	29 (9.0)	63 (7.7)	18 (9.4)
Speedisk® C18	MeOH	–	–	89 (4.2)	79 (8.9)	84 (5.0)	85 (8.9)
	EtAc	–	–	40 (5.7)	50 (8.8)	83 (9.7)	80 (7.0)
	ACN	–	–	49 (10)	63 (9.4)	74 (7.7)	70 (5.8)

Neither the extraction column nor the eluent used did work well for the TBPA. Further studies provided the explanation. TBPA in water samples was quite quickly hydrolysed into tetrabromophthalic acid that has higher polarity than that of TBPA and was eluted in the void time. Therefore, TBPA could not be retained on SPE columns from water samples. Data analysis from Table 4 indicates that the best eluent, which gave the highest recoveries on each column (except Speedisk®) for most of the compounds, was EtAc. The Speedisk® did not retain TBPP, and the Oasis HLB and the Bond Elut Nexus columns gave recoveries under 35 % for some FRs and were eliminated from further studies.

The columns that provided the best recoveries for FRs were then tested with 1 L of spiked (2 μg of TPhP, TBBPA and EHDP and 5 μg of TBPA, TBPP and HBCD) distilled water samples to confirm their recoveries. The selected columns were as follows: the Bond Elut PPL, ENV and C18LO, which recovered more than 70 % for most of the compounds. The recoveries obtained in 1 L of spiked distilled water are presented in Table 5.

**Table 5:** Recoveries for FRs using ethyl acetate (*n* = 3)

Extraction column	Recoveries [%] (% RSD)				
	TBPP	TPhP	TBBPA	EHDP	HBCD
Bond Elut ENV	101 (9.5)	92 (4.1)	76 (3.7)	91 (5.0)	82 (3.2)
Bond Elut PPL	90 (8.1)	82 (5.5)	73 (4.1)	83 (8.0)	63 (7.5)
Bond Elut C18LO	94 (6.6)	98 (5.9)	55 (10)	78 (3.2)	73 (4.8)

Most of the obtained recoveries were at the same level as in distilled water. The column that provided the best recoveries for all of the compounds was the Bond Elut ENV column. The recovery levels were over 80 % for all FRs except TBBPA (76 %). It is worth adding that the satisfactory precision was achieved for all extraction columns, even though the absolute recoveries were lower for PPL and C18LO. The Bond Elut ENV column, though, was selected for the analysis of environmental samples from rivers and lakes. The water sample Zrodlo Karola from Ustron (Poland) was selected to confirm the recoveries in surface water samples. None of the FRs were found in those sample, and recoveries achieved using the extraction procedure with the ENV column were as follows: 95 % (TBPP), 96 % (TPhP), 83 % (TBBPA), 89 % (EHDP) and 86 % (HBCD).

### Linearity, Method Detection Limit, Method Quantification Limit and Intra- and Inter-day Precision

Standard curves were determined using linear regression *y* = *ax* + *b*, where *y* is the peak area, *a* is the slope, *x* is the respective concentration, and *b* is the intercept. The exact parameters of the obtained calibration curves were calculated (Table 6). The method detection limit (MDL) and method quantification limit (MQL) values were determined using the parameters of the obtained standard curves. The MDL was calculated as MDL = 3.3*s*/*a*, where *s* is the standard deviation of the blank samples and *a* is the slope. The MQL value was determined as MQL = 3MDL. The MDL and MQL values obtained by this method were recalculated including the appropriate recovery level of each drug in 1 L of surface water. Therefore, the calculated values included all steps introduced to the analytical procedure. For most of the FRs, MQL values were under 1 μg L^−1^. As a result, we conclude that they can be detected in surface waters when present.

**Table 6:** Parameters of calibration curves, linearity ranges and MDL and MQL values

Drug	Linear range [μg L^−1^]	Slope (*a*)	*S*_*a*_	Intercept (*b*)	*S*_*b*_	*S*_*xy*_	*R* ^2^ (*n* = 6)	MDL [μg L^−1^]	MQL [μg L^−1^]
TBPP	1.75–25	559	32	−330	426	606	0.9937	0.518	1.555
TPhP	0.03–20	48,521	2,371	38,637	24,472	37,535	0.9952	0.008	0.023
TBBPA	0.25–20	82,130	2,194	96,528	25,164	31,188	0.9986	0.081	0.244
EHDP	0.50–20	27,572	398	22,937	22,937	6,073	0.9996	0.144	0.431
HBCD	1.00–50	1,004	5	−106	129	204	0.9999	0.317	0.949

Method intra- and inter-day precision was evaluated by testing at two concentration levels (2 and 20 μg L^−1^). The intra-day precision was calculated at days 1 and 7, and inter-day precision was calculated between days 1 and 7 using the same method. The values obtained are presented in Table 7.

**Table 7:** Intra- and inter-day precision

Analyte	Intra-day precision (RSD%) (*n* = 6)				Inter-day precision (RSD%) (*n* = 12)	
	Day 1		Day 7		Days 1–7	
	2 μg mL^−1^	20 μg mL^−1^	2 μg mL^−1^	20 μg mL^−1^	2 μg mL^−1^	20 μg mL^−1^
TBPP	7.91	6.08	5.71	6.25	10.83	9.52
TPhP	5.43	6.38	6.87	5.76	12.12	8.34
TBBPA	4.97	5.07	7.41	8.88	10.20	8.93
EHDP	6.15	7.37	9.25	8.02	13.89	7.18
HBCD	8.95	6.20	7.15	7.10	11.00	4.02

### Environmental Samples

A variety of water samples from the Vistula River (Ustron), the Bytomka River (Zabrze), the Klodnica River (Gliwice), Bagier Lake (Zabrze), Kokotka Lake (Ruda Slaska), Marcina Lake (Ruda Slaska), SEMAG Lake (Zabrze), Pileckiego Lake (Zabrze) and Smrodlok Lake (Ruda Slaska) were tested. One-litre samples were pre-concentrated on the Bond Elut ENV columns using the SPE procedure.

Some of the flame retardants were found in water samples as presented in Table 8. The FRs in water samples were identified by the comparison of the retention of standard solutions and by addition of detected analyte to the extract, in order to check the retention time and peak shape.

**Table 8:** Concentrations and standard deviations (μg L^−1^) of FRs in different surface water samples (*n* = 3)

Water samples	Concentration (SD) [μg L^−1^]				
	TBPP	TPhP	TBBPA	EHDP	HBCD
The Vistula River, Ustron	–	–	–	–	–
The Bytomka River, Zabrze	–	–	0.30 (0.08)	0.49 (0.11)	–
The Klodnica River, Gliwice	–	0.30 (0.07)	0.49 (0.10)	0.47 (0.09)	–
Bagier Lake, Zabrze	–	–	–	–	1.33 (0.21)
Kokotka Lake, Ruda Slaska	–	0.12 (0.03)	–	–	2.15 (0.34)
Marcina Lake, Ruda Slaska	–	–	–	–	2.89 (0.26)
SEMAG Lake, Zabrze	–	–	0.41 (0.09)	–	–
Pileckiego Lake, Zabrze	–	0.03 (0.01)	0.26 (0.05)	0.45 (0.08)	3.10 (0.27)
Smrodlok Lake, Ruda Slaska	–	–	–	0.48 (0.07)	2.75 (0.38)

Relatively high concentrations (over 1.0 μg L^−1^) of HBCD were found in some lakes. This could be caused by the presence of industrial facilities in the nearest locations that recycle the plastic materials. The rest of the FRs were determined in relatively low concentrations near theirs MQL levels.

**Fig. 3:**
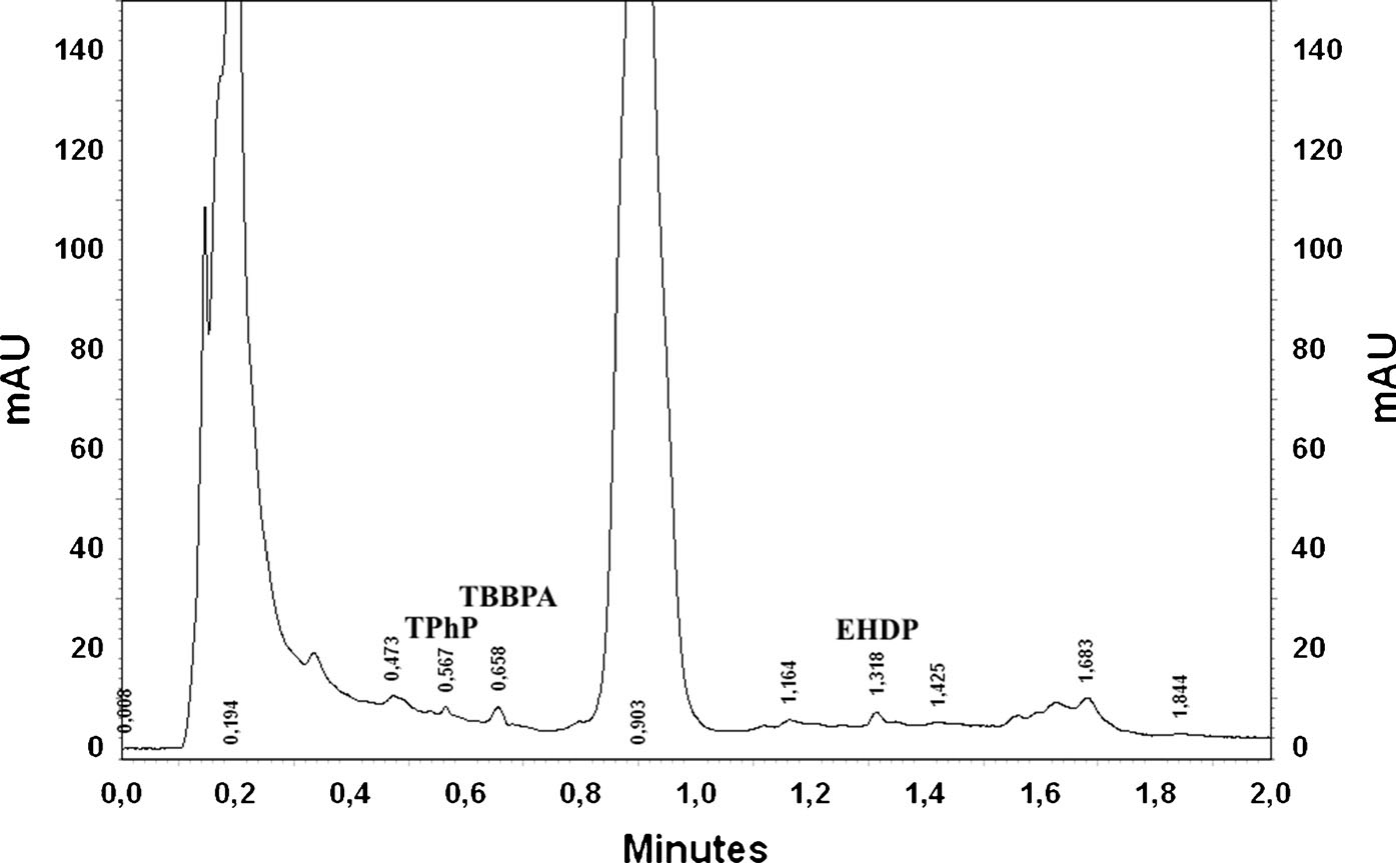
The chromatogram of the Klodnica River after SPE procedure

**Fig. 4:**
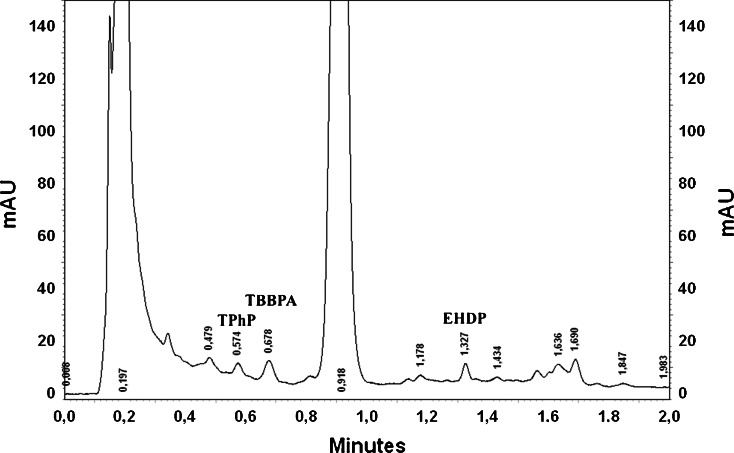
The chromatogram of the Klodnica River with addition of 1 μg TPhP, TBBPA and 0.5 μg EHDP

The chromatogram of the Klodnica River after the SPE procedure is presented in Fig. 3. As can be seen, some peaks were present, but only three of them could be quantified (after the addition of standard). The remaining peaks are unidentified (Fig. 4). All of the investigated FRs were added to the sample, but none of the existing peaks could be identified as one of those FRs.

## Conclusions

A new, very fast and sensitive method has been developed for the determination of flame retardants in water samples using ultra-HPLC equipment and UV detection. All FRs can be determined with good separation and within 2 min using this chromatographic system.

The combination of a fast UHPLC-UV system together with an optimised SPE technique is selective, efficient and precise and can be used for different water samples. All compounds studied can be pre-concentrated from 1-L water samples with very high recoveries (over 80 % in surface water samples) using Bond Elut ENV columns. Low values of MDL and MQL as well as wide linear range and good intra- and inter-day precision of the developed method are satisfactory for the determination of FRs. Therefore, this method with optimised extraction procedure was used for the analysis of surface water samples from rivers and lakes. Applying the procedure, some of FRs were found, mostly in low concentrations—under 0.5 μg L^−1^. However, relatively high concentrations of HBCD could be very dangerous for the environment. Thus, the concentration of FRs should be monitored, especially in those waters that could enter into drinking water systems.

To conclude, the optimised SPE pre-concentration procedure and the ultra-HPLC method for determination of the selected FRs have been successfully applied to the analysis of surface water samples and can be used as a monitoring tool for FRs in water samples.
